# Majority of the erythrocyte binding proteins of the Pvfam “a” family of *Plasmodium vivax* interact with Basigin to assist parasite entry into the host cell

**DOI:** 10.3389/fcimb.2025.1592281

**Published:** 2025-06-30

**Authors:** Manish Tripathi, Meghna Santoshi, Yagya D. Sharma, Sumit Rathore

**Affiliations:** Department of Biotechnology, All India Institute of Medical Sciences, New Delhi, India

**Keywords:** vivax malaria, erythrocyte receptor, red cell invasion, protein–protein interactions, parasite growth inhibition

## Abstract

Molecular mechanisms of red cell invasion by the *Plasmodium vivax* parasite remain obscure since information on receptor–ligand interaction is scarce. Several proteins of the *P. vivax* Pvfam “a” family are known to bind with host erythrocytes. Some of them share their erythrocyte receptors with each other and *vice versa*, but the identification of these receptors is awaited with the exception of PvTRAg38. Here, we demonstrate by using solid-phase binding assay and surface plasmon resonance that majority (7 out of 10) of these erythrocyte binding proteins (PvTRAg, PvTRAg33.5, PvTRAg35.2, PvTRAg34, PvTRAg36, PvTRAg38, and PvTRAg69.4) interact with the erythrocyte receptor Basigin. These interactions seem to be important for the parasite’s survival since each of these proteins interfered with the parasite’s growth in a heterologous culture system. Furthermore, a higher parasite growth inhibition rate was observed with the combination of these proteins, suggesting the significance of multiple parasite ligand’s interaction with the same erythrocyte receptor during the invasion process. These results will be helpful in understanding *P. vivax* biology and developing the therapeutics for vivax malaria.

## Introduction

Every year, a large proportion of the human population suffers from malaria caused by *Plasmodium vivax*. This parasite, besides *Plasmodium falciparum*, is very common in Southeast Asian and South American countries. However, the molecular mechanisms of this parasite involved in host–parasite interaction are scarcely known. In this regard, the Duffy antigen receptor for chemokines has been proposed to be involved in the red cell invasion by *P. vivax* merozoites where this receptor interacts with the Duffy binding protein of the parasite (Miller et al., 1976; Chitnis and Miller, 1994; Kanjee et al., 2021). However, recent literature gives an indication that there are additional receptor–ligand interactions involved in the red cell invasion process because Duffy-negative humans were also found to be infected with this parasite (Menard et al., 2010; Popovici et al., 2020). Some of these additional receptors and their ligands have been identified, such as CD71-PvRBP2b (Gruszczyk et al., 2018), CD98hc (Malleret et al., 2021), Basigin-PvTRAg38 (Rathore et al., 2017), Band 3-PvTRAg38 (Alam et al., 2015), Band 3-PvTRAg36 (Alam et al., 2016a; Alam et al., 2016b), and Band 3-PvTRAg74 (Alam et al., 2016a). Since several other parasite ligands also bind to host erythrocytes, their interaction with the respective erythrocyte receptors need to be explored to understand the parasite’s biology and to develop therapeutics against the disease.

It is known that several parasite proteins of the Pvfam “a” family, also known as PvTRAgs (*P. vivax* tryptophan-rich antigens), are highly immunogenic with conserved sequences in parasite population and bind to host erythrocytes (Jalah et al., 2005; Garg et al., 2007; Alam et al., 2008; Garg et al., 2008). This erythrocyte binding activity was inhibited by the respective PvTRAg antibodies, purified from the patients’ sera, indicating their immunobiological significance during *P. vivax* infection (Mittra et al., 2010; Zeeshan et al., 2013). The respective erythrocyte receptors for these PvTRAgs are not yet identified, with the exception of the above-mentioned few. Here, we describe that the majority of erythrocyte binding PvTRAgs recognize Basigin as their erythrocyte receptor, and blocking of this receptor with these PvTRAgs inhibits the parasite growth.

## Materials and methods

### Ethics statement

For parasite culture, heparinized venous blood was collected from healthy donors above 18 years of age (*n* = 10) visiting the department, after a written consent. Blood was collected following the institutional ethical guidelines. The Ethics Committee of All India Institute of Medical Sciences, New Delhi, had approved the study via approval number IECPG-532/26.10.2016.

### Materials

RPMI 1640, hypoxanthine, penicillin–streptomycin, fetal calf serum, glutamine, glutaraldehyde, HBS-EP buffer (degassed and ready to use 0.01 M HEPES, pH 7.4, 0.15 M NaCl, 3 mM EDTA, and 0.005% v/v Surfactant P20) (Cytiva 100 Results Wy, Marlborough, MA 01752, USA, Cat No. BR100826), MAC magnet separation column (Macs; Miltenyi Biotec), and E64 (Cat No. E3132 Sigma). Recombinant histidine-tagged PvTRAgs, i.e., PvTRAg (Sarin and Sharma, 2006), PvTRAg33.5, PvTRAg35.2, PvTRAg34, PvTRAg36, PvTRAg69.4, PvTRAg36.6, PvTRAg26.3, PvTRAg74 (Zeeshan et al., 2015), and PvTRAg38 (Rathore et al., 2017), bacterial (*Desulfovibrio desulfuricans*) thioredoxin (Rathore et al., 2017), and human Basigin (Rathore et al., 2017) were available in the lab from previous studies. Bacterial thioredoxin has been utilized as negative control in the interaction studies as has been done earlier (Rathore et al., 2017).

### Solid-phase binding assay

A 96-well microtiter plate was coated with 50 nM of histidine-tagged individual PvTRAg or histidine-tagged bacterial thioredoxin in carbonate buffer (pH 9.6) and incubated overnight at 4°C. The coated enzyme-linked immunosorbent assay (ELISA) plate was blocked with 5% bovine serum albumin (BSA) in phosphate-buffered saline (PBS) for 2 h at 37°C. The different concentrations of histidine-tagged recombinant Basigin [0–3.2 μM (Malleret et al., 2021)] were added to these wells and plates were incubated for 2 h at 37°C. Plates were washed with PBST (PBS containing 0.05% Tween 20) and incubated with 1:2,000 dilution of the primary rabbit polyclonal anti-Basigin antibody, followed by horseradish peroxidase (HRP)-conjugated anti-rabbit IgG secondary antibody (Pierce, Cat. No. 31460). Finally, plates were developed with o-phenyldiamine substrate, and O.D. was measured at 490 nm.

### Surface plasmon resonance

This assay was done on an Autolab Esprit Instrument (Eco Chemie, Utrecht, The Netherlands) at 25°C, using HBS buffer (10 mM HEPES + 100 mM NaCl and 2 mM EDTA, pH 7.4). The recombinant Basigin (1 μM) was immobilized on a CM5 sensor chip using the amine-coupling method. Kinetic analysis was then performed by flowing different concentrations of the analyte (PvTRAg, PvTRAg36, PvTRAg33.5, PvTRAg35.2, PvTRAg69.4, PvTRAg34, or PvTRAg38) on the immobilized Basigin as well as reference flow cell, followed by regeneration with 50 mM NaOH at the end of each cycle. The association kinetics was monitored for 400 s followed by dissociation for the next 100 s. Reference-subtracted sensograms were analyzed using in-built software in the Autolab Esprit Instrument. The binding constant, *K*_D_, was calculated as *k*_d_/*k*_a_ using data analysis software, and kinetic rate constants were determined by fitting the corrected response data to a simple 1:1 Hill-Langmuir binding isotherm model.

### Plasmodium falciparum culture and growth inhibition assay

Growth inhibition assay was performed as described by Boyle et al (Boyle et al., 2010). Briefly, the *P. falciparum* 3D7 strain was cultured in complete RPMI 1640 medium containing 0.5 g/L Albumax I, 27.2 mg/L hypoxanthine, and 2 g/L sodium bicarbonate, using O^+^ human erythrocytes obtained from healthy donors (4% hematocrit) under mixed gas (5% O_2_, 5% CO_2_, and 90% N_2_). Late-stage parasites (40–46 h after invasion) were isolated (>95% purity) from infected red blood cells (RBCs) with a MAC separation column (Macs; Miltenyi Biotec). Purified schizonts were washed using RPMI and incubated for 6 to 8 h with 10 μM E-64 to get completely mature segmented schizonts. Schizonts were pelleted at 2,000 rpm for 5 min and washed thrice in RPMI. The parasites were resuspended in 5 mL of culture media at room temperature and filtered through a 2-μm-pore-size disc 25-mm syringe filter. Merozoites were collected by centrifugation, suspended by pipetting gently, and introduced into wells containing RBCs with different concentrations (0–20 µM) of PvTRAg, PvTRAg33.5, PvTRAg35.2, PvTRAg34, PvTRAg36, and PvTRAg69.4, PvTRAg38, or PvTRAg38.7 in a 96-well culture plate in triplicate. Uninfected erythrocytes, infected erythrocytes alone, and infected erythrocytes with PBS were taken as controls. Furthermore, parasites were maintained for 3–6 h and stained with ethidium bromide. A total of 100,000 events were acquired per sample, using Cell Quest software on a FACS Caliber flow cytometer (Becton Dickenson Biosciences, Palo Alto, CA, USA).

### Statistical analysis

All the obtained data shown are being analyzed using one-way analysis of variance (ANOVA). Mean ± SD values have been used to plot the graph.

## Results

### Human Basigin interacts with majority of erythrocyte binding PvTRAgs of Pvfam “a” family

**Figure 1:**
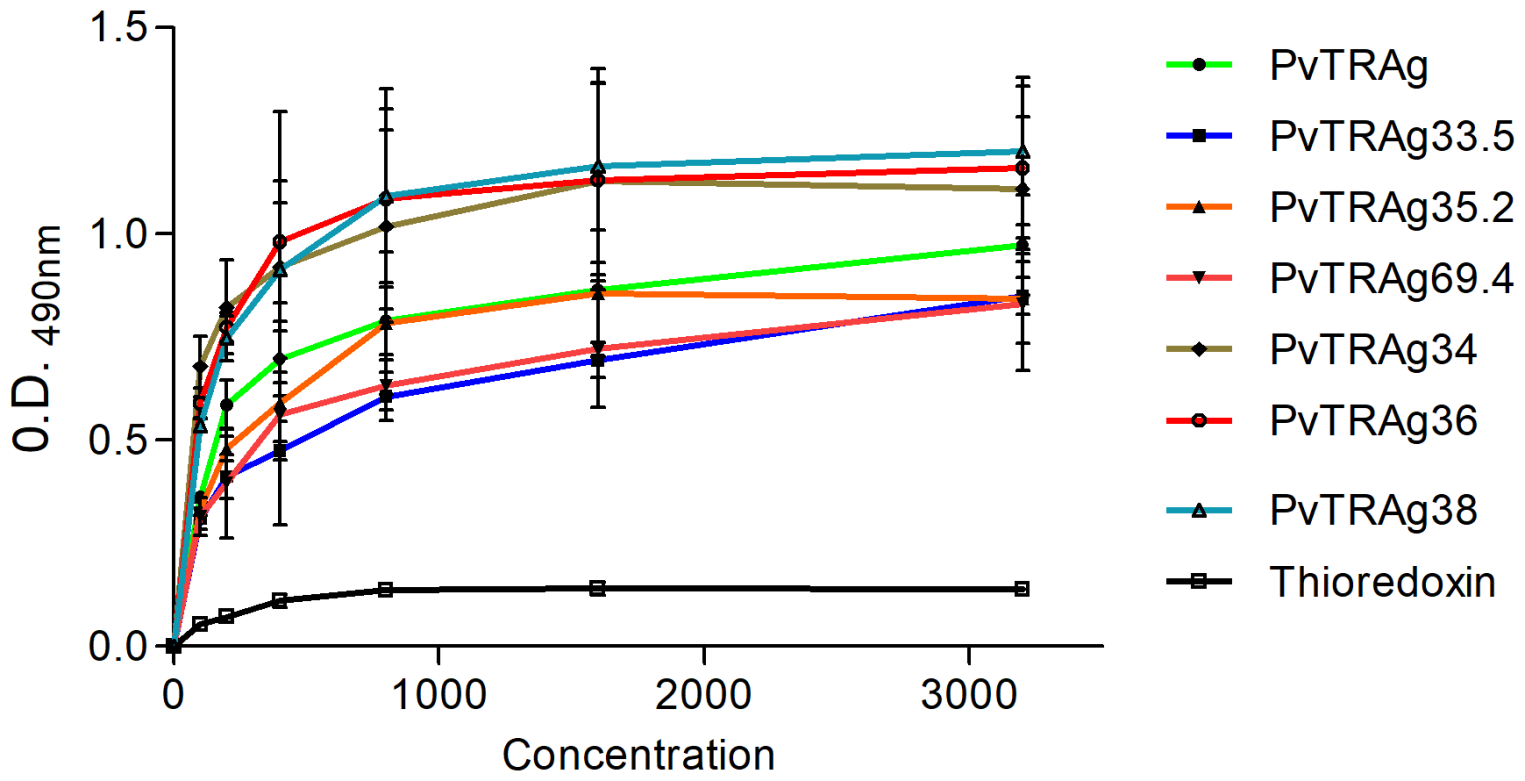
Binding of Basigin to Pvfam “a” family proteins. Solid-phase binding assay. Increasing concentrations (0–3.2 µM) of different PvTRAgs were added to the wells of an ELISA plate already coated with 50 nM histidine-tagged Basigin or histidine-tagged bacterial thioredoxin. The plate was developed with anti-Basigin polyclonal antibody as described in the text. Mean ± SD value of absorbance from three experiments is plotted.

In order to find out which PvTRAg(s) recognize the erythrocyte receptor Basigin, we carried out the solid-phase binding assay using Basigin and all the 10 proteins of the Pvfam “a” family that had earlier been reported to bind to host erythrocytes (Tyagi and Sharma, 2012). The results showed that 7 out of these 10 proteins, i.e., PvTRAg, PvTRAg33.5, PvTRAg35.2, PvTRAg34, PvTRAg36, PvTRAg69.4, and PvTRAg38, showed interaction with human Basigin in a concentration-dependent manner (**Figure 1**). Indeed, PvTRAg38 was used here as a positive control as it has already been reported to bind with Basigin (Rathore et al., 2017). The remaining three PvTRAgs (namely, PvTRAg36.6, PvTRAg26.3, and PvTRAg74) did not show interaction with Basigin as they did not show a concentration-dependent increase in O.D. values, which remained similar to that of the negative control, bacterial thioredoxin. Binding of these PvTRAgs to Basigin was further confirmed by the surface plasmon resonance (SPR) data (**Table 1**). They showed that binding affinity (*K*_D_ values) with Basigin ranged between 1.25 × 10^−6^ M and 5.1 × 10^−6^ M.

**Table 1:** Affinity of different PvTRAgs with Basigin based on surface plasmon resonance assay.

Name of protein	*K*_D_ value
PvTRAg	1.5 ± 0.8 × 10^−6^
PvTRAg33.5	5.1 ± 1.1 × 10^−6^
PvTRAg36	1.3 ± 0.2 × 10^−6^
PvTRAg35.2	1.75 ± 0.7 × 10^−6^
PvTRAg69.4	1.95 ± 0.1 × 10^−6^
PvTRAg38	1.3 ± 0.2 × 10^−6^
PvTRAg 34	1.25 ± 0.4 × 10^−6^

### All PvTRAgs showing interaction with Basigin also interfere with the parasite growth

**Figure 2:**
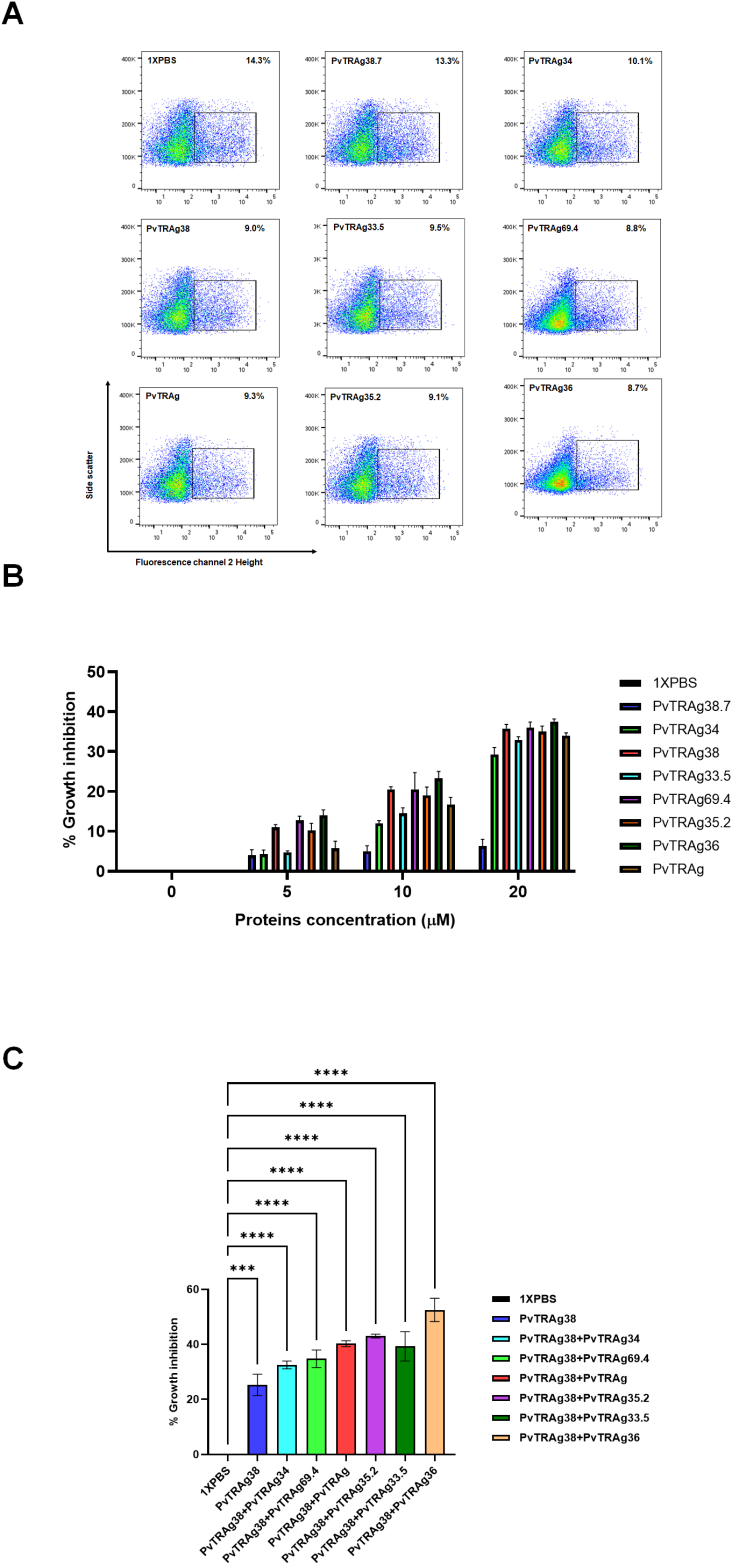
Inhibition of *P. falciparum* growth by PvTRAgs. Purified merozoites were incubated with PvTRAg38, PvTRAg33.5, PvTRAg35.2, PvTRAg69.4, PvTRAg, PvTRAg34, PvTRAg36, PvTRAg38PvTRAg38.7, and other controls. Parasitemia was determined by ethidium bromide staining and measured by flow cytometry. **(A)** Representative dot plots showing parasitemia after treatment with different PvTRAgs at a concentration of 20 µM. **(B)** Bar diagram shows the percentage of parasite growth inhibition at different concentrations of PvTRAgs. Data shown are the mean ± SD for two triplicate experiments. **(C)** Bar diagram showing the parasite growth inhibition with different PvTRAgs combination (10 µM each). Data shown are the mean ± SD for two triplicate experiments (*p* < 0.0001). *** = *p* <0.001, ****=*p* <0.0001.

Since *P. vivax* is difficult to culture, we have used here the heterologous *P. falciparum* culture system to study parasite growth inhibition, as described earlier (Alam et al., 2015; Rathore et al., 2017). This is because Basigin was not shed off during the maturation of reticulocytes and continues to be present on mature RBCs, which are used by the *P. falciparum* merozoites for invasion. Results showed that all of the above-mentioned seven PvTRAgs, including PvTRAg38, were able to inhibit the parasite growth (**Figure 2A**). Their potential of parasite growth inhibition varied from 28% to 38% at 20 μM concentration. The negative control PvTRAg38.7, which does not bind to erythrocytes, did not show any remarkable effect on the parasite growth. There was a dose-dependent effect of each PvTRAg on the parasite growth inhibition rate (**Figure 2B**). Furthermore, the addition of another protein along with PvTRAg38 to the culture further reduced the parasite growth as compared to a single protein, e.g., PvTRAg38 + PvTRAg34 (~32%), PvTRAg38 + PvTRAg69.4 (~34%), PvTRAg38 + PvTRAg (~40%), PvTRAg38 + PvTRAg35.2 (~43%), PvTRAg38 + PvTRAg33.5 (~39%), and PvTRAg38 + PvTRAg36 (~52.5%) (**Figure 2C**). The maximum rate of parasite growth inhibition was observed with the combination of PvTRAg36 and PvTRAg38.

## Discussion

Our earlier cross-competition studies have revealed that several PvTRAgs compete with each other for erythrocyte binding, thereby indicating that they were sharing their erythrocyte receptor(s) (Zeeshan et al., 2015). This was indeed evident from another report where the Band 3 receptor on the host erythrocyte has been shown to recognize three different proteins of the Pvfam “a” family, namely, PvTRAg38, PvTRAg36, and PvTRAg74 (Alam et al., 2016b). Partial abolition of PvTRAg38 binding activity with the chymotrypsin-treated erythrocytes was also an indication that this parasite ligand recognized more than one erythrocyte receptor (Tyagi and Sharma, 2012). Later studies identified Basigin as the second erythrocyte receptor, besides Band 3, for this parasite ligand (Alam et al., 2015). Since PvTRAg38 was cross-competing, partially or fully, with several other PvTRAgs, we decided to investigate if this second erythrocyte receptor was also being recognized by these proteins. The solid-phase binding and SPR assay results of the present study indeed showed that majority of the erythrocyte binding PvTRAgs (7 out of 10) of the Pvfam “a” family (PvTRAg, PvTRAg33.5, PvTRAg35.2, PvTRAg34, PvTRAg36, PvTRAg69.4, and PvTRAg38) bind to this host erythrocyte receptor Basigin (**Figure 1**). It is quite surprising that so many parasite proteins are interacting with the same erythrocyte receptor, although it is not unusual for the parasite to utilize multiple ligands to recognize the same host receptor and *vice versa* (Paing and Tolia, 2014).

What could be the implication of this receptor–ligand interaction phenomenon for the parasite’s biology? For this, we planned to investigate if these additional six PvTRAgs, besides PvTRAg38, that interact with Basigin are also able to interfere with the parasite’s growth. Since *P. vivax* is difficult to maintain in *in vitro* culture, we have used a heterologous *P. falciparum* culture system. This was based on the fact that the Basigin receptor also plays an important role in *P. falciparum* merozoite invasion of RBCs albeit using a different parasite ligand (Williams et al., 2012). In our earlier studies, we used it to study parasite growth inhibition due to the blockade of the Basigin receptor with PvTRAg38 (Rathore et al., 2017). Indeed, all seven PvTRAgs, including PvTRAg38, were able to inhibit parasite growth (**Figure 2A**). These results indicate that all of the seven proteins recognizing Basigin were involved in red cell invasion. Thus, interaction of these seven proteins of the Pvfam “a” family plays an important role in the parasite’s biology during the red cell invasion process.

Why did the parasite develop such a complex system of receptor–ligand interaction where each receptor is recognized by multiple parasite ligands and *vice versa* for the host cell invasion process? To address this question, partly, we used different combinations of proteins to observe their effect on parasite growth. Results showed that the addition of any of the six proteins to PvTRAg38 in the culture significantly reduced parasite growth (**Figure 2C**). This suggests that the parasite may be using multiple proteins to bind to the same erythrocyte receptor for a stronger interaction between receptor and ligand to ensure an effective parasite entry into the host cell for the invasion process. Such an additive effect of combination of PvTRAgs on parasite growth could possibly be occurring due to their interaction with each other and then effectively blocking the receptor.

## Data Availability

The original contributions presented in the study are included in the article/supplementary material. Further inquiries can be directed to the corresponding author.

## References

[B1] Alam M. T. Bora H. Singh N. Sharma Y. D. (2008). High immunogenecity and erythrocyte-binding activity in the tryptophan-rich domain (TRD) of the 74-kDa Plasmodium vivax alanine-tryptophan-rich antigen (PvATRAg74). Vaccine 26, 3787–3794. doi: 10.1016/j.vaccine.2008.05.059 18579264

[B2] Alam M. S. Choudhary V. Zeeshan M. Tyagi R. K. Rathore S. Sharma Y. D. (2015). Interaction of plasmodium vivax tryptophan-rich antigen pvTRAg38 with band 3 on human erythrocyte surface facilitates parasite growth. J. Biol. Chem. 290, 20257–20272. doi: 10.1074/jbc.M115.644906 26149684 PMC4536434

[B3] Alam M. S. Rathore S. Tyagi R. K. Sharma Y. D. (2016a). Host-parasite interaction: multiple sites in the Plasmodium vivax tryptophan-rich antigen PvTRAg38 interact with the erythrocyte receptor band 3. FEBS Lett. 590, 232–241. doi: 10.1002/1873-3468.12053 26823170 PMC7163959

[B4] Alam M. S. Zeeshan M. Rathore S. Sharma Y. D. (2016b). Multiple Plasmodium vivax proteins of Pv-fam-a family interact with human erythrocyte receptor Band 3 and have a role in red cell invasion. Biochem. Biophys. Res. Commun. 478, 1211–1216. doi: 10.1016/j.bbrc.2016.08.096 27545606

[B5] Boyle M. J. Wilson D. W. Richards J. S. Riglar D. T. Tetteh K. K. Conway D. J.. (2010). Isolation of viable Plasmodium falciparum merozoites to define erythrocyte invasion events and advance vaccine and drug development. Proc. Natl. Acad. Sci. United States America 107, 14378–14383.1 doi: 10.1073/pnas.1009198107 PMC2922570 20660744

[B6] Chitnis C. E. Miller L. H. (1994). Identification of the erythrocyte binding domains of Plasmodium vivax and Plasmodium knowlesi proteins involved in erythrocyte invasion. J. Exp. Med. 180, 497–506. doi: 10.1084/jem.180.2.497 8046329 PMC2191600

[B7] Garg S. Alam M. T. Das M. K. Dev V. Kumar A. Dash A. P.. (2007). Sequence diversity and natural selection at domain I of the apical membrane antigen 1 among Indian Plasmodium falciparum populations. Malar J. 6, 154. doi: 10.1186/1475-2875-6-154 18031585 PMC2211494

[B8] Garg S. Chauhan S. S. Singh N. Sharma Y. D. (2008). Immunological responses to a 39.8kDa Plasmodium vivax tryptophan-rich antigen (PvTRAg39.8) among humans. Microbes Infect. 10, 1097–1105. doi: 10.1016/j.micinf.2008.05.008 18603013

[B9] Gruszczyk J. Kanjee U. Chan L. J. Menant S. Malleret B. Lim N. T. Y.. (2018). Transferrin receptor 1 is a reticulocyte-specific receptor for Plasmodium vivax. Science 359, 48–55. doi: 10.1126/science.aan1078 29302006 PMC5788258

[B10] Jalah R. Sarin R. Sud N. Alam M. T. Parikh N. Das T. K.. (2005). Identification, expression, localization and serological characterization of a tryptophan-rich antigen from the human malaria parasite Plasmodium vivax. Mol. Biochem. Parasitol. 142, 158–169. doi: 10.1016/j.molbiopara.2005.01.020 15869815

[B11] Kanjee U. Gruring C. Babar P. Meyers A. Dash R. Pereira L.. (2021). Plasmodium vivax strains use alternative pathways for invasion. J. Infect. Dis. 223, 1817–1821. doi: 10.1093/infdis/jiaa592 32941614 PMC8161644

[B12] Malleret B. El Sahili A. Tay M. Z. Carissimo G. Ong A. S. M. Novera W.. (2021). Plasmodium vivax binds host CD98hc (SLC3A2) to enter immature red blood cells. Nat. Microbiol. 6, 991–999. doi: 10.1038/s41564-021-00939-3 34294905

[B13] Menard D. Barnadas C. Bouchier C. Henry-Halldin C. Gray L. R. Ratsimbasoa A.. (2010). Plasmodium vivax clinical malaria is commonly observed in Duffy-negative Malagasy people. Proc. Natl. Acad. Sci. United States America 107, 5967–5971. doi: 10.1073/pnas.0912496107 PMC2851935 20231434

[B14] Miller L. H. Mason S. J. Clyde D. F. McGinniss M. H. (1976). The resistance factor to Plasmodium vivax in blacks. The Duffy-blood-group genotype, FyFy. N Engl. J. Med. 295, 302–304. doi: 10.1056/NEJM197608052950602 778616

[B15] Mittra P. Singh N. Sharma Y. D. (2010). Plasmodium vivax: immunological properties of tryptophan-rich antigens PvTRAg 35.2 and PvTRAg 80.6. Microbes Infect. 12, 1019–1026. doi: 10.1016/j.micinf.2010.07.004 20638483

[B16] Paing M. M. Tolia N. H. (2014). Multimeric assembly of host-pathogen adhesion complexes involved in apicomplexan invasion. PloS Pathog. 10, e1004120. doi: 10.1371/journal.ppat.1004120 24945143 PMC4055764

[B17] Popovici J. Roesch C. Rougeron V. (2020). The enigmatic mechanisms by which Plasmodium vivax infects Duffy-negative individuals. PloS Pathog. 16, e1008258. doi: 10.1371/journal.ppat.1008258 32078643 PMC7032691

[B18] Rathore S. Dass S. Kandari D. Kaur I. Gupta M. Sharma Y. D. (2017). Basigin interacts with plasmodium vivax tryptophan-rich antigen pvTRAg38 as a second erythrocyte receptor to promote parasite growth. J. Biol. Chem. 292, 462–476. doi: 10.1074/jbc.M116.744367 27881677 PMC5241724

[B19] Sarin R. Sharma Y. D. (2006). Thioredoxin system in obligate anaerobe Desulfovibrio desulfuricans: Identification and characterization of a novel thioredoxin 2. Gene 376, 107–115. doi: 10.1016/j.gene.2006.02.012 16580795

[B20] Tyagi R. K. Sharma Y. D. (2012). Erythrocyte binding activity displayed by a selective group of plasmodium vivax tryptophan rich antigens is inhibited by patients’ Antibodies. PloS One 7, e50754. doi: 10.1371/journal.pone.0050754 23236392 PMC3516511

[B21] Williams A. R. Douglas A. D. Miura K. Illingworth J. J. Choudhary P. Murungi L. M. (2012). Enhancing blockade of Plasmodium falciparum erythrocyte invasion: assessing combinations of antibodies against PfRH5 and other merozoite antigens. PLoS Pathog. 8(11), e1002991. doi: 10.1371/journal.ppat.1002991 23144611 PMC3493472

[B22] Zeeshan M. Bora H. Sharma Y. D. (2013). Presence of memory T cells and naturally acquired antibodies in Plasmodium vivax malaria-exposed individuals against a group of tryptophan-rich antigens with conserved sequences. J. Infect. Dis. 207, 175–185. doi: 10.1093/infdis/jis650 23087432

[B23] Zeeshan M. Tyagi R. K. Tyagi K. Alam M. S. Sharma Y. D. (2015). Host-parasite interaction: selective Pv-fam-a family proteins of Plasmodium vivax bind to a restricted number of human erythrocyte receptors. J. Infect. Dis. 211, 1111–1120. doi: 10.1093/infdis/jiu558 25312039

